# Family Planning in Zambia: An Investment Pillar for Economic Development

**DOI:** 10.12688/gatesopenres.12989.2

**Published:** 2020-07-27

**Authors:** Michael T. Mbizvo, Nicole Bellows, Joseph G. Rosen, Stephen Mupeta, Chisha A. Mwiche, Ben Bellows

**Affiliations:** 1 Population Council, Lusaka, Zambia; 2 Avenir Health, Washington, District of Columbia, USA; 3 United Nations Population Fund, Lusaka, Zambia; 4 Ministry of Health, Government of the Republic of Zambia, Lusaka, Zambia; 5 Population Council, Washigton, District of Columbia, USA

**Keywords:** family planning, FP2020, sustainable development goals, social-economic development, costed implementation plan, Zambia

## Abstract

Family planning represents a ‘best buy’ in global efforts to achieve sustainable development and attain improvements in sexual and reproductive health. By meeting contraceptive needs of all women, significant public health impact and development gains accrue. At the same time, governments face the complex challenge of allocating finite resources to competing priorities, each of which presents known and unknown challenges and opportunities. Zambia has experienced a slow but steady increase in contraceptive prevalence, with slight decline in total fertility rate (TFR), over the past 20 years. Drawing from the Zambian context, including a review of current policy solutions, we present a case for making investments in voluntary family planning (FP), underpinned by a human rights framework, as a pillar for accelerating development and socio-economic advancement. Through multilevel interventions aimed at averting unintended pregnancies, Zambia – and other low- and middle-income countries – can reduce their age dependency ratios and harness economic growth opportunities awarded by the demographic dividend while improving the health and quality of life of the population.

## Abbreviations

DHS: Demographic and Health Survey, FP: Family planning, GRZ: Government of the Republic of Zambia, SSA: Sub-Saharan Africa, SDGs: Sustainable Development Goals, UNESCO: United Nations Educational, Scientific and Cultural Organization, UNFPA: United Nations Population Fund, TFR: Total fertility rate

## Disclaimer

The views expressed in this article are those of the authors. Publication in Gates Open Research does not imply endorsement by the Gates Foundation.

## Introduction

Among the goals set by the global community for sustainable development is Goal 3.7, **“** By 2030 ensure universal access to sexual and reproductive health care services, including family planning (FP) information and education, and the integration of reproductive health into national strategies and programmes ^1^. ”Meeting all contraceptive and maternal and newborn health care needs would result in substantial health and development gains, yielding dramatic reductions of 76% in unintended pregnancy, 74% in less-than-safe and unsafe abortions, 64% in maternal deaths, and 76% in newborn deaths ^2^. Investments in FP, therefore, offer benefits beyond fertility, further downstream of the maternal and newborn care continuum.

Low-income countries with high fertility rates are vulnerable to poor maternal and child health outcomes, economic stagnation, environmental degradation, and political unrest ^3^. These outcomes trap societies in a vicious poverty cycle, with women bearing a disproportionate burden. By making investments that expand access to a broad contraceptive method mix, which includes both short- and long-acting contraception, based on principles of voluntarism and informed choice, countries can generate economic benefits while also improving the health, education, and quality of life for current and future populations ^4^.

Investments in high-quality FP services yield increased uptake and continued use of modern contraception, which have positive impacts on the health of women and children ^5^. Healthier women with fewer children participate more in the workplace, resulting in economic benefits that also extend to children who are better educated and equipped to be more economically productive in the future ^4^. When children are healthier, and infant mortality declines, the average number of desired children decreases, increasing demand for FP and unlocking a virtuous cycle for continuing improvements in health and economic advancement ^6^.

Additionally, when population dynamics shift due to a reduction in mortality and fertility rates, there is a resulting increase in the proportion of working-age adults ^7^. When combined with appropriate policies, including the expansion of FP through increased availability and accessibility of contraceptive options (including long-acting methods like intrauterine devices and hormonal implants), this shift can yield the ‘demographic dividend’, a phenomenon in which there are relatively fewer dependents (children and elderly) and a greater proportion of working-age adults, spurring rapid economic development and income generation ^7^. This phenomenon has been observed in East Asia and certain regions of South America but has yet to be harnessed in most of sub-Saharan Africa (SSA), where FP uptake has been lower, and the HIV/AIDS epidemic has affected many working-age adults ^7^. Realization of the demographic dividend in SSA is possible, but this opportunity can be missed or greatly diminished without proper investments in FP. As Melinda Gates stated in the Gates Foundation 2017 Annual Letter, “No country in the last 50 years has emerged from poverty without expanding access to contraceptives ^8^.”

## Economic estimates of FP investments

The results of research on the impact of FP vary widely across settings and intervention contexts; nonetheless, a systematic review demonstrated that cross-cutting investments in women’s health yield healthier and more productive societies, broadly ^6^. One seminal study in Bangladesh found that investment in community-based FP services had a positive and persistent long-term contribution in targeted localities 19 years later, with a faster decline in fertility, a lower birth rate, reductions in infant and child mortality, healthier women with 40% higher wages, and increased educational gains among their children ^5^.

Another systematic review found that scaling FP interventions was the most cost-effective strategy for reducing maternal mortality ^9^. In a review of global progress and strategies to reduce maternal mortality, contraceptive use emerged as a chief factor associated with substantial declines in maternal mortality ^10^.

Other cost–benefit and economic analyses of FP interventions have attributed substantial economic advantages to FP investments. The Guttmacher Institute’s 2014 “Adding It Up” report estimated that every $1 (USD) spent on contraception would generate $1.47 in savings from averting unwanted pregnancies ^10^. Another estimate showed very high benefit-to-cost ratios associated with voluntary FP programs, ranging from 90 to 150, meaning that the costs can potentially yield over 100 times the cost in benefits, when accounting for the economic gains from lower infant and maternal mortality and higher income growth ^11^. The same report discussed the post-2015 consensus that policymakers should prioritize the elimination of unmet need for modern contraception by 2040 ^11^.

Economic models in Africa have predicted substantial gains if fertility rates can accelerate their decline. One simulation model in Nigeria found that shifting the fertility rate from the UN median fertility projection to the low fertility projection would result in a 5.6% increase in per capita income within 20 years and an 11.2% increase in 50 years ^12^. In Ghana, an analysis with four simulations of prospective government investment estimated that across funding scenarios, the health savings from public sector investments in FP would exceed costs associated with service provision in absence of those investments, offering further evidence to include fee-for-service reimbursement of FP services in the national insurance plan ^13^.

By describing reproductive health challenges and innovative policy strategies in the Zambian context, we present a case for investments in voluntary FP, underpinned by a human rights framework, as a pillar for accelerating development and socio-economic advancement. Through multilevel interventions aimed at averting unintended pregnancies, Zambia – and other low- and middle-income countries – can reduce their age dependency ratios and harness economic growth opportunities awarded by the demographic dividend while improving the health and quality of life of the population.

## Demographic and FP trends: Zambia case study

In 2018, Zambia had a total fertility rate (TFR) of approximately 4.7 births per woman, a substantial drop from 6.5 births in 1992, with rural women having an average of twice as many children as urban women (5.8 vs. 3.4 births) ^14^. In spite of high fertility in Zambia, there have been substantial gains in the use of modern contraception in recent years, with 48% of married women of reproductive age reporting use of some form of modern contraception, an increase from only 15% in 1992 ^14, 15^.

Despite these advancements, challenges addressing the demand for FP in Zambia persist. Data from the 2018 Zambia Demographic and Health Survey (DHS) reveal that 20% of women of childbearing age have an unmet need for FP, meaning that one-fifth of these women wish to prevent or delay childbearing but are not using any form of FP ^14^. In addressing unmet need, efforts to expand FP access may focus either on the concerns and needs of women who have never used or women who discontinued use due to stock-outs, side-effects, partner preferences, or other reasons ^16^. A third (36%) of Zambian women reported discontinuing a contraceptive method within one year of initiation ^14^. Injectables and oral contraceptives, both combined estrogen-progestin and progestin-only, account for 67% of modern methods used among Zambian women ^14^. While user preferences contribute to method uptake and continuation patterns, supply-side barriers – including stock-outs of preferred methods and limited method mix (one to two methods offered in health facilities) – strain uptake and sustained use of modern contraception. Being able to switch to another method enables women to maintain protection from pregnancy, but this requires access to a broad mix of methods, from shorter-acting methods to longer-acting reversible contraceptives ^17^.

In addition to having high levels of unmet need, Zambian women commence childbearing early, with one-third reporting their first birth before their 18 ^th^ birthday and more than half by age 20 ^14^. Among teenaged girls (ages 13–19), 29% have begun childbearing ^14^. While child marriage in Zambia has declined in recent years, over one-third of women aged 20–49 reported being married before age 18, one of the highest rates in the world ^14^. Geographic analysis of child marriage in Zambia identified Northern, Muchinga, and Copperbelt Provinces as having the highest rates of child marriage; however, girls in Western and North-Western Provinces were more likely to experience an adolescent pregnancy ^18^.

The culmination of early childbearing and unmet need for FP contribute to a high birth rate in Zambia, at 40 per 1,000 population per year, by contrast with the worldwide average of 19 per 1,000 and an average of 37 per 1,000 for SSA ^19^. As such, Zambia has a higher age dependency ratio, or proportion of the population that are children or elderly. While the worldwide age dependency ratio is 54%, it is 85% in SSA and 95% in Zambia ^19^. Increased investments in voluntary FP could yield reductions in this ratio and advance economic growth.

At present, the United Nations median estimate for Zambia suggests 553,000 women did not have their FP needs met in 2017; this estimate is projected to increase to 609,000 women by 2030 ^20^. As a consequence, many women are at risk of unintended pregnancy. In 2016, an estimated 443,000 unintended pregnancies were recorded in Zambia, but 352,000 were averted due to use of modern contraception ^21^. By further increasing the number of averted unintended pregnancies in the coming years, Zambia is well positioned to lower its age dependency ratio and harness the economic growth opportunities awarded by the demographic dividend, while improving the health and quality of life of the population.

### Current efforts and recommendations

The Government of the Republic of Zambia (GRZ) has been actively working to generate demand for, expand dialogue on, and improve access to FP in a coordinated effort guided by the national costed implementation plan ^21^. This plan emerged in response to the 2012 London Summit on Family Planning, where the government articulated several commitments to improving contraceptive outcomes by 2020, including a commitment to increasing voluntary FP access for those in need by doubling its budget for FP commodities and enhancing community-based outreach, with the goal of reaching a modern contraceptive prevalence rate of 58% ^21^. As of August 2016, GRZ reported substantial progress in securing FP commodities and building provider capacity, as well as engaging traditional and religious leaders in dialogues surrounding child marriage and adolescent pregnancy ^21^. GRZ is near the end of an 8-year (2013–2020) FP scale-up plan focused on strengthening demand for FP, engaging adolescents and rural women in addition to improving service delivery and government coordination ^21^.

Looking ahead to 2030, GRZ has committed to the Sustainable Development Goals (SDGs), which build on the Millennium Development Goals and focus on sustainability and root causes of poverty. Many of the 17 SDGs are linked to key metrics in sexual and reproductive health including FP access, connecting these indicators to improvements in health, gender equality, education, and sustainability ^21^. For example, SDG 3.7 aims to “ensure universal access to sexual and reproductive health care services, including for family planning, information and education, and the integration of reproductive health into national strategies and programmes ^1^.”

Moving forward with these commitments, GRZ must maintain focus on the unique opportunities and challenges of FP provision in Zambia. First, GRZ’s focus on adolescents and rural women is critical, since these groups have historically lagged in contraceptive uptake. Current strategies to engage adolescents include continued support of comprehensive sexuality education in schools through capacity strengthening of teachers and peer educators, identifying youth-friendly service access points and address existing stigma at health facilities inhibiting youth FP uptake, and clarifying age of consent regulations to improve youth access ^22^.

Sustained integration of FP and HIV services nationally will further mitigate geographic access barriers, particularly for underserved populations in rural areas. The recently completed Evidence for Contraceptive Options and HIV Outcomes (ECHO) Trial showed high HIV incidence in women across study arms, defined by allocation to an FP formulation (i.e., injectable, implant, or copper intrauterine device) ^23^. These incidence trends underscore potential inadequacies with current implementation of HIV risk assessments in FP service provision. Comprehensive HIV risk assessments are vital clinical decision-making tools, shaping client and provider interactions around FP recommendations, and conduits to complementary health services, including antiretroviral therapy for HIV-positive women and pre-exposure prophylaxis for at-risk women. Strengthening the frequency and quality of these assessments can help close existing service-delivery gaps and lead to more responsive FP service provision. 

In addition to FP demand generation and securing FP commodities for these sub-populations, it is also important to combine FP efforts with other policy interventions, particularly those aimed at reducing early marriage and promoting school retention for girls longer than the current average of 4.3 years ^14^. In addition to reducing the TFR, delaying first birth can yield substantial socio-economic benefits by enabling more women to access education and earn higher wages ^4^. With support from the United Nations Population Fund (UNFPA) and the United Nations Economic, Scientific and Cultural Organization (UNESCO), GRZ has developed and launched an adolescent sexual and reproductive health curriculum for grades 5-12 in public schools. The curriculum leverages trained peer educators to foster dialogue and cultivate awareness of salient sexual and reproductive health and rights issues confronting adolescents, from contraception to healthy relationships with peers and caregivers to pregnancy and sexually transmitted infections ^21^.

Efforts to address the 20% of women with an unmet need for FP will require not only reaching those who have never used FP previously, but also those who have used at least one FP method but later discontinued use. By expanding the mix of methods available to women, particularly for longer-acting reversible methods, end users can find the method that works best for them, wherever they may be in their reproductive trajectories, thereby minimizing the risk of discontinuation and poor adherence. Additionally, a broader method mix enhances choice, a core component of a rights-based approach to FP outreach and provision. According to the 2018 DHS, over a third of births to Zambian women were not desired at that time or ever ^14^. Proposed policy solutions for tackling this unmet need for FP, articulated in GRZ’s costed FP scale-up plan, include enhanced staff training for integrating long-acting contraceptive methods into routine FP provision and task-shifting contraceptive delivery to community-based distributors ^21^. Through expanded access to a range of short- and long-acting contraception, women’s FP preferences can be realized, alongside substantial improvements in health, as well as education and economic development.

From a public administrative perspective, it is important to both protect FP resources in government budgeting and to increase domestic investment in provision of voluntary FP services. Specifying that allocated funds are for FP supplies and provision helps to improve budgeting transparency; without specification, line item funds risk being absorbed into broader maternal and child health expenses. Increasing domestic investment in voluntary FP services is critical at a time when donor assistance in FP is stagnant or even declining ^24^. Itemizing contraceptive commodities and services and advocating for increased domestic FP investment, ministries, and the future national health insurance fund can increase security of planned FP commodity procurements and lead to subsequent reductions in the financial burden of pregnancy-related costs ^10, 25^. Bolstering FP governance, through subnational coordination of national FP initiatives and establishment of novel tracking systems for procurements and expenditures, should be a core tenant of efforts to safeguard FP commodity security and sustain access to a mix of contraceptive methods. Finally, as a public administrative goal, it is important to work with local leaders to ensure ownership of FP objectives and promote a common goal of improving health, economic, and human welfare.

## Conclusions

Decades of research have clearly established the benefits of voluntary FP services as a health, development, and human rights priority. It has a direct impact on women’s health and socio-economic development. There is a pressing need to invest more in voluntary FP services in general and, specific to the Zambian context, to realize the development potential associated with high and sustained contraceptive use. This requires the engagement of diverse stakeholders, economic and planning decision-makers, as well as traditional and religious leaders. With proper planning and investments in FP and related supportive programs, Zambia is at a critical juncture in harnessing the demographic dividend, spurring long-term economic growth and continued improvements in the population’s health, education, and overall quality of life. Unfortunately, this opportunity comes at a time when international resources for FP investments are under threat, and the COVID-19 pandemic threatens to disrupt FP supply chains and service provision. Therefore, it is important to also mobilize domestic resources for FP investments and continue to explore innovative strategies to increase FP access for women with current unmet needs, especially in the current pandemic context. Ensuring a broad contraceptive method mix secures decision-making autonomy over reproductive intentions for women and couples. Ultimately, this will require commitments to FP programs and policies by government officials across line ministries, as well as greater public support.

## Data availability

No data are associated with this article.
