# Supplementary material for: Family Planning in Zambia: An Investment Pillar for Economic Development
Source: Gates Open Res. 2020 Jul 27;3:1459. Originally published 2019 May 8. [Version 2] doi: 10.12688/gatesopenres.12989.2 (PMC7429446; doi:10.12688/gatesopenres.12989.2)
Supplement: Peer review history

# Peer review: Reviewer response for version 2

**DOI:** 10.21956/gatesopenres.14364.r29229

**Published:** 2020-08-13

**Authors:** John Stanback

**Affiliations:** 1 FHI 360, Durham, NC, USA

I thank the authors and congratulate them on this new and improved version of their manuscript.

Does the article adequately reference differing views and opinions?

Yes

Are all factual statements correct, and are statements and arguments made adequately supported by citations?

Yes

Is the Open Letter written in accessible language?

Yes

Where applicable, are recommendations and next steps explained clearly for others to follow?

Partly

Is the rationale for the Open Letter provided in sufficient detail?

Partly

Reviewer Expertise:

programmatic family planning researcher with >30 years experience

I confirm that I have read this submission and believe that I have an appropriate level of expertise to confirm that it is of an acceptable scientific standard.

# Peer review: Reviewer response for version 2

**DOI:** 10.21956/gatesopenres.14364.r29230

**Published:** 2020-08-03

**Authors:** Mai Do

**Affiliations:** 1 Department of Global Community Health and Behavioral Sciences, Tulane University School of Public Health and Tropical Medicine, New Orleans, LA, USA

I believe that the authors have responded to the comments thoroughly and adequately.

Does the article adequately reference differing views and opinions?

Yes

Are all factual statements correct, and are statements and arguments made adequately supported by citations?

Yes

Is the Open Letter written in accessible language?

Yes

Where applicable, are recommendations and next steps explained clearly for others to follow?

Yes

Is the rationale for the Open Letter provided in sufficient detail?

Yes

Reviewer Expertise:

15 years of experience in family planning and reproductive health.

I confirm that I have read this submission and believe that I have an appropriate level of expertise to confirm that it is of an acceptable scientific standard.

# Peer review: Reviewer response for version 1

**DOI:** 10.21956/gatesopenres.14096.r28485

**Published:** 2020-02-06

**Authors:** Mai Do

**Affiliations:** 1 Department of Global Community Health and Behavioral Sciences, Tulane University School of Public Health and Tropical Medicine, New Orleans, LA, USA

I agreed with the first reviewer that this is an excellent, very well written open letter with many clear policy implications. I only have two comments:

  

1. While the authors aim to use Zambia to make a case for investments in FP, I am left wanting to know more about Zambia to be convinced that Zambia is such a good example. This is because when the authors describe current efforts in Zambia, they do a very good job in the first half. In the second half, when they start talking about reducing early marriage, promoting education, expanding method mix, etc, it is not clear what the Government of Zambia has done exactly. No specific examples from the country’s programs are provided.
2. The abstract is too heavy on the background, with the first three sentences saying the same key points. Later on, the sentence “Increasing voluntary modern contraceptive use…” in the middle of the abstract is similar to the last sentence of the abstracts as they both talk about socioeconomic outcomes and demographic dividends. Both of these instances give an impression that the abstract could be much more concise and to the point.

Does the article adequately reference differing views and opinions?

Yes

Are all factual statements correct, and are statements and arguments made adequately supported by citations?

Yes

Is the Open Letter written in accessible language?

Yes

Where applicable, are recommendations and next steps explained clearly for others to follow?

Yes

Is the rationale for the Open Letter provided in sufficient detail?

Yes

Reviewer Expertise:

15 years of experience in family planning and reproductive health.

I confirm that I have read this submission and believe that I have an appropriate level of expertise to confirm that it is of an acceptable scientific standard, however I have significant reservations, as outlined above.

# Response

**Published:** 2020-07-20

**Authors:** Joseph Rosen

**Affiliations:** Population Council, Zambia

We thank the reviewer for these thoughtful, detailed comments. In response to the first point about specific policies implemented in Zambia, we have clarified our language in the “Current efforts and recommendations” sub-section (Pgs. 6-7) in hopes of delineated between current FP policies implemented in Zambia and our recommendations. Additionally, at the reviewer’s recommendation, we have supplemented our discussion of policy recommendations with additional detail of existing FP policies in Zambia, particularly those enumerated in the costed implementation plan to support FP scale-up (2013-2020). These include development of a school-based adolescent sexual and reproductive health curriculum and task-shifting FP provision to community-based distributors. With respect to the second comment, we have consolidated some of the language in the abstract (Pg. 2) to more succinctly summarize of the Open Letter’s contents.

# Peer review: Reviewer response for version 1

**DOI:** 10.21956/gatesopenres.14096.r28057

**Published:** 2019-10-25

**Authors:** John Stanback

**Affiliations:** 1 FHI 360, Durham, NC, USA

This is an excellent open letter: well-written, concise and with clear policy implications. My only issues are with a couple of significant omissions.  

 First, another benefit of family planning is that it helps countries become more resilient, not only in the face of climate change, but also to other shocks and emergencies.

 Second, and more significantly, I was very disappointed that, in a letter full of precise and direct policy recommendations, the authors seemed extremely hesitant to directly address the need for more long-acting methods such as implants and IUDs. Instead, they use roundabout wording to signal this lacuna, citing the problem of early method discontinuation and the need for a broader method mix. If you are serious about improving choice, please be more explicit. You've covered your bases by mentioning "rights" five times and "voluntary" ten times in a single letter; it's okay to say "long-acting" at least once.

Does the article adequately reference differing views and opinions?

Yes

Are all factual statements correct, and are statements and arguments made adequately supported by citations?

Yes

Is the Open Letter written in accessible language?

Yes

Where applicable, are recommendations and next steps explained clearly for others to follow?

Partly

Is the rationale for the Open Letter provided in sufficient detail?

Partly

Reviewer Expertise:

programmatic family planning researcher with >30 years experience

I confirm that I have read this submission and believe that I have an appropriate level of expertise to state that I do not consider it to be of an acceptable scientific standard, for reasons outlined above.

# Response

**Published:** 2020-07-20

**Authors:** Joseph Rosen

**Affiliations:** Population Council, Zambia

We thank the reviewer for the thorough and thoughtful review of our Open Letter. We agree with the reviewer’s assertion that FP supports women’s resilience to confront climate change, natural disasters, and other destabilizing events. As such, we have added language to the Open Letter’s introduction (Pg. 3) addressing these aforementioned factors as added benefits to investments in FP. Secondly, we also appreciate the reviewer’s attention to the absence of explicit articulation of long-acting methods in our discussion of FP. To correct this oversight, we have weaved discussion of long-acting methods throughout our manuscript, including explicit mention of long-acting methods as a core component of the recommendation for expanding contraceptive method mix.
